# From Genome to Phenome: Genotype × Environment Interactions in Organic and Conventional Dairy Systems and the Emergence of Genomically Optimized Organic Dairy (GOOD)

**DOI:** 10.3390/genes17090990

**Published:** 2026-08-24

**Authors:** Priunka Bhowmik, Amy Zinski, Qingqing Wu, Weiwei Du, Jennifer J. Michal, Ramanathan Kasimanickam, Zhihua Jiang

**Affiliations:** 1 Department of Animal Sciences, Washington State University, Pullman, WA 99164-7620, USA; priunka.bhowmik@wsu.edu (P.B.); amy.zinski@wsu.edu (A.Z.); qingqing.wu@wsu.edu (Q.W.); wdu@wsu.edu (W.D.); jennifer_michal@wsu.edu (J.J.M.); 2 Department of Veterinary Clinical Sciences, Washington State University, Pullman, WA 99164-7060, USA; ramkasi@wsu.edu

**Keywords:** organic and conventional dairy, genotypes × environment interaction, genomics and phenomics, animal and human health

## Abstract

Organic dairy farming has expanded rapidly over the past three decades, driven by regulatory reforms, consumer demand, and growing recognition of its environmental, animal welfare, and potential human health benefits. Despite this growth, evidence comparing organic and conventional dairy systems remains fragmented across genetics, phenomics, animal health, and human health outcomes. This review synthesizes current knowledge through the lens of genotype × environment interactions, integrating evidence from four complementary domains: (1) genomic architecture and breeding strategies; (2) phenotypic performance, including milk production and composition, meat quality, nutrition, and reproductive traits; (3) animal health, disease resistance, antimicrobial use, and welfare; and (4) implications for human health. Holstein–Friesian cattle remain the predominant breed in both systems; however, organic production favors animals with greater robustness, longevity, grazing efficiency, and disease resilience. Genetic studies further demonstrate that highly heritable production traits share similar genetic architecture across production systems, whereas health, fertility, longevity, and other low-heritability functional traits exhibit stronger genotype × environment interactions and more system-specific genomic signatures. These findings suggest that breeding strategies developed for high-input conventional systems are unlikely to maximize performance under organic management. Collectively, the evidence supports a shift from selection focused primarily on milk yield toward genomic improvement of robustness, disease resistance, reproductive resilience, grazing adaptation, and lifetime productivity. We propose Genomically Optimized Organic Dairy (GOOD) as an emerging framework that integrates genomic selection, precision phenotyping, health monitoring, and environmental adaptation to develop dairy cattle better suited to organic production.

## 1. Introduction

**Figure 1:**
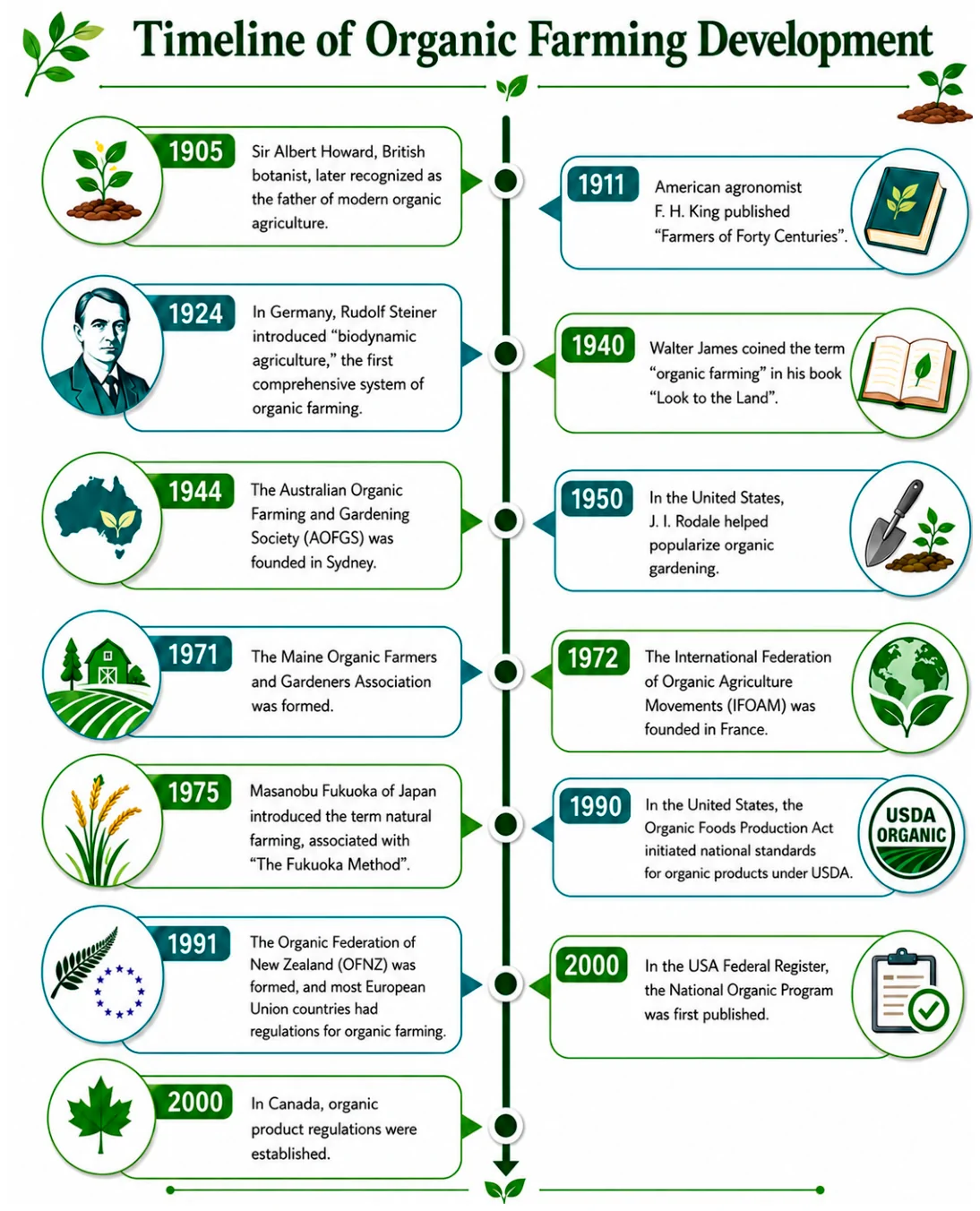
Timeline of organic agriculture worldwide.

Organic agriculture emerged in the early twentieth century as a response to the increasing industrialization of farming and the growing dependence on synthetic fertilizers, pesticides, and external inputs. Its conceptual foundation was shaped by early thinkers such as Sir Albert Howard, who emphasized soil fertility, composting, and the biological integrity of agroecosystems, and F. H. King, who documented sustainable traditional farming systems in Farmers of Forty Centuries [1,2]. Rudolf Steiner’s biodynamic agriculture, Walter James’s use of the term “organic farming,” J. I. Rodale’s promotion of organic gardening in the United States, and Masanobu Fukuoka’s natural farming philosophy further strengthened the ecological and low-input principles of organic agriculture [3,4,5,6,7]. These ideas later became institutionalized through the Organic Foods Production Act of 1990, the USDA National Organic Program, and national organic regulations in countries such as the United States and Canada [8,9,10] (Figure 1). Since the early 2000s, consumer demand for environmentally sustainable, animal-welfare-oriented, and residue-conscious food systems has accelerated the expansion of organic agriculture, including organic dairy production [11].

Organic dairy production emerged as a specialized branch of the organic movement and became prominent during the 1990s, driven by consumer concerns about food safety, environmental sustainability, animal welfare, and biotechnology in animal production, particularly after the commercial introduction of recombinant bovine growth hormone in the United States [12]. Early organic dairy farms relied more on pasture, on-farm resources, and reduced external inputs than conventional systems, and despite efficiency improvements in both systems, they remain distinct in regulation, management priorities, and biological context [13,14,15]. Before unified national standards, organic dairy operated under fragmented private and state-level certification systems, while later market coordination by companies such as Organic Valley and Horizon Organic helped commercialize and structure the organic dairy sector [16].

Regulation is central to defining organic dairy systems. In the United States, the USDA National Organic Program, administered by the USDA Agricultural Marketing Service, establishes the standards for certified organic dairy production [17,18]. These standards require dairy cattle to receive 100% certified organic feed, obtain at least 30% of their dry matter intake from pasture during the grazing season, and graze for a minimum of 120 days per year [19,20]. Organic certification also prohibits synthetic growth hormones, genetically modified organisms, and routine antibiotic use [19,20] (Table S1). In contrast, conventional dairy systems generally allow greater flexibility in feed formulation, therapeutic intervention, reproductive management, housing, and production intensification. Therefore, organic and conventional dairy systems should not be viewed simply as different marketing categories, but as distinct production environments that expose cows to different nutritional, managerial, health, and selection pressures.

**Figure 2:**
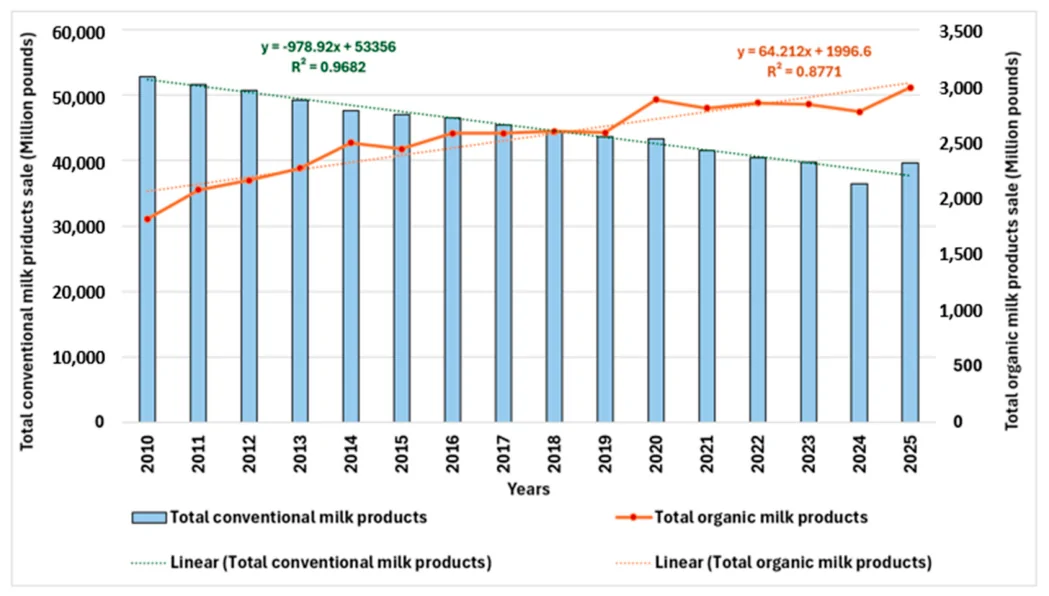
Total organic and conventional milk products sales in the USA (2010–2025).

These biological and management differences are reflected in divergent production and market trends, with USDA data showing that the U.S. organic dairy cow population increased from 2265 animals in 1992 to 352,289 in 2021, representing approximately 3.8% of the national dairy herd [21]. From 2010 to 2025, conventional milk product sales declined from 52.8 to 39.7 billion pounds, whereas organic milk product sales increased from 1.81 to 2.98 billion pounds [22,23,24,25,26,27,28,29,30,31,32,33,34,35,36,37] (Figure 2), indicating a structural shift toward growing consumer interest in organic dairy, a trend also projected globally due to concerns about animal welfare, pesticide and antibiotic use, and environmental sustainability [38]. However, organic dairy producers continue to face major constraints, including higher feed and certification costs, restricted antibiotic therapy, pasture-compliance requirements, disease-control challenges, and limited breeding values tailored to low-input production systems.

The central biological distinction between organic and conventional dairy systems is that they expose the same animal genome to different production environments. Conventional systems generally emphasize high milk yield through energy-dense diets, intensive management, high-producing genotypes, and production-oriented breeding, whereas organic systems prioritize pasture access, forage-based nutrition, reduced synthetic inputs, prevention-based health management, and animal adaptability. These contrasting environments can influence milk yield, fertility, disease susceptibility, welfare, longevity, and milk composition. Pasture-based organic diets may increase milk omega-3 fatty acids and reduce omega-6 fatty acid proportions, although composition is also affected by breed, season, diet, and lactation stage [39,40]. Because nutrition, disease exposure, management intensity, and selection pressure differ between systems, genotype-by-environment interactions may occur, meaning that animals selected for superior performance in conventional high-input systems may not express the same genetic merit under organic or pasture-based conditions. Consistent with this possibility, Shabalina et al. [41] reported differences in estimated genetic parameters and associated loci.

Although previous studies have compared specific aspects of organic and conventional dairy systems, evidence remains fragmented across genetics, productivity, reproduction, health, milk quality, and human health outcomes. Therefore, this review synthesizes current knowledge on organic and conventional dairy systems through the lens of biological variation and genotype-by-environment interaction. Specifically, it examines how divergent production environments shape dairy cow performance, disease susceptibility, reproductive efficiency, milk quality, and genomic selection response, while proposing the emergence of Genomically Optimized Organic Dairy as a future framework for integrating genome, phenome, and health data across production environments.

## 2. Genomic Responses to Production Environments: Insights into Genotype × Environment Interactions

### 2.1. Breeds, Lines and Breeding Systems

Breed composition and performance. Breed composition in organic dairy farming is generally similar to that of conventional dairy systems, with Holstein–Friesian cattle remaining the predominant breed in many countries, including Germany, Canada, and Spain [42]. However, the extent of Holstein dominance varies across countries, and organic systems in some regions maintain a greater representation of local breeds. For example, Swedish organic herds contain a higher proportion of Swedish Red cattle (54.3%) and a lower proportion of Holstein–Friesians (35.5%) than conventional herds, where Holsteins account for 46.9% of cows. Likewise, in Switzerland and Austria, local breeds such as Brown Swiss and Fleckvieh are widely used in both conventional and organic systems, while Dutch organic farms often incorporate native breeds alongside Holsteins [42]. These findings suggest that although breed diversity in organic farming broadly mirrors that of conventional production, organic systems in some countries place greater emphasis on locally adapted breeds.

Differences in breed composition between organic and conventional systems are partly related to the distinct management environments and breeding objectives of the two production systems. Organic dairy farming typically relies more heavily on grazing, forage-based feeding, and restricted veterinary interventions, with greater emphasis on animal health, fertility, longevity, and adaptability rather than maximizing milk output [43]. As a result, breeds that perform well under lower-input conditions may be favored in organic systems, particularly in countries where local breeds have historically been selected for functional traits and environmental adaptation.

Studies conducted under organic production conditions consistently show a trade-off between milk yield and functional performance among breeds. In Germany, organic Original Angler cows produced less energy-corrected milk than German Holsteins (5193 vs. 5620 kg) but had higher milk fat (5.09% vs. 4.18%) and protein contents (3.61% vs. 3.31%) and tended to have fewer days open [44]. Similarly, in Sweden, Swedish Holsteins achieved the highest milk yield (9209 kg), whereas Swedish Red and Swedish Polled cattle produced less milk but had higher milk fat and protein concentrations and superior fertility characteristics, including fewer days open and, for Swedish Polled cattle, fewer inseminations per conception [44]. Swedish Red cows also exhibited lower rates of veterinary treatments, particularly for udder health disorders, indicating advantages in functional health traits.

Evidence comparing breed performance across production systems further supports these patterns. Using Swedish dairy populations, Clasen et al. [45] reported that Swedish Red cows produced approximately 5–11% less energy-corrected milk than Swedish Holsteins in both conventional and organic systems. However, Swedish Red cattle generally exhibited superior fertility and health, including lower risks of mastitis, metritis, displaced abomasum, and mortality, as well as improved conception rates and reproductive efficiency, regardless of the production system. Collectively, these studies indicate that Holstein cattle maintain a clear advantage in milk production regardless of production system, whereas local breeds tend to demonstrate superior functional traits such as fertility, health, and milk component quality. These advantages may be particularly valuable under organic management, where breeding goals extend beyond milk yield to include robustness and adaptation to low-input production environments [42,44,45].

Holstein lines and performance. Within the Holstein breed, different genetic lines have been developed under contrasting production environments, with North American- and European-derived Holsteins generally selected for high milk production in more intensive conventional systems, whereas New Zealand Holstein–Friesians have been bred under pasture-based, lower-input conditions that resemble many organic production systems. The suitability of these lines for organic farming has attracted attention because organic systems rely heavily on grazed forage and limited concentrate supplementation. In a comparison of Swiss Holstein–Friesian (HCH) and New Zealand Holstein–Friesian (HNZ) cows managed in an organic grazing system without concentrate supplementation, both lines achieved similar milk yields and herbage intake, indicating that the high-production Swiss Holstein strain was unable to fully express its genetic potential under the nutrient constraints of an organic pasture-based system [46]. This finding suggests that line differences observed under conventional, high-input conditions may be reduced in organic systems where nutrient availability is more limited.

Despite similar milk yield, the two Holstein strains differed in several functional and physiological traits under organic management. New Zealand Holstein–Friesian cows produced milk with higher fat (4.05% vs. 3.69%) and protein concentrations (3.20% vs. 2.92%) and exhibited higher circulating concentrations of IGF-1 and triiodothyronine (T3), indicating a greater ability to cope with restricted nutrient supply in grazing systems [46]. In contrast, Swiss Holsteins showed greater organic matter digestibility but spent less time ruminating. A subsequent study found no significant differences between strains in milk production, pasture intake, nutrient digestibility, or energy expenditure under organic grazing conditions, despite Swiss Holsteins spending more time grazing and performing more grazing mastication activities [47]. Overall, these results indicate that Holstein strains selected in pasture-based environments may possess functional adaptations that are advantageous under organic management, whereas strains developed for intensive conventional systems do not necessarily achieve superior performance when concentrate supplementation is restricted.

Purebreeding vs. crossbreeding. Under organic dairy production, crossbreeding has generally been associated with improvements in functional traits while maintaining competitive production performance. In organically managed herds in northern Spain, Holstein–Friesian cows produced the highest milk yield, whereas Brown Swiss cows produced the lowest, with crossbred cows occupying an intermediate position for milk production [48]. However, crossbred cows achieved the highest fat yield and equal or greater protein yield compared with the pure breeds, resulting in energy-corrected milk (ECM) yields that were comparable to or higher than those of Holsteins, particularly in older cows. Crossbred cows also exhibited superior reproductive performance, requiring fewer services per conception, achieving higher conception rates at first service, and maintaining shorter calving-to-conception and calving intervals than both Holstein–Friesian and Brown Swiss cows. In addition, crossbreds showed favorable calving performance, with the highest proportion of unassisted calvings and the lowest incidence of difficult births, and had lower culling rates due to lameness and death or urgent slaughter than Holsteins. These findings suggest that crossbreeding under organic conditions can improve fertility, calving ease, and survivability while maintaining satisfactory milk production [48].

Evidence from studies comparing breeding strategies across both organic and conventional systems indicates that the advantages of crossbreeding extend beyond organic production. Using Swedish Holstein and Swedish Red populations, Clasen et al. [45] demonstrated that crossbreeding improved profitability in both systems, primarily through enhanced fertility, health, and longevity rather than increased milk yield. Compared with purebred Holstein herds, crossbred herds exhibited higher conception rates, shorter calving intervals, lower replacement rates, reduced cow and calf mortality, and lower incidence of several health disorders. Milk production remained similar between purebreeding and terminal crossbreeding systems and declined only slightly (1–2%) under rotational crossbreeding. These improvements translated into higher economic returns in both organic and conventional herds. Complementary evidence from Pereira and Heins [49] further suggests that breed composition influences behavioral traits under both organic grazing and low-input conventional management. Although overall activity levels were similar between Holsteins and crossbreds, rumination behavior differed among breed groups, indicating potential differences in feed utilization and adaptation to low-input environments. Collectively, these studies demonstrate that crossbreeding can enhance functional efficiency, reproductive performance, and herd sustainability in both organic and conventional dairy systems while largely preserving production levels.

### 2.2. Heritability

**Figure 3:**
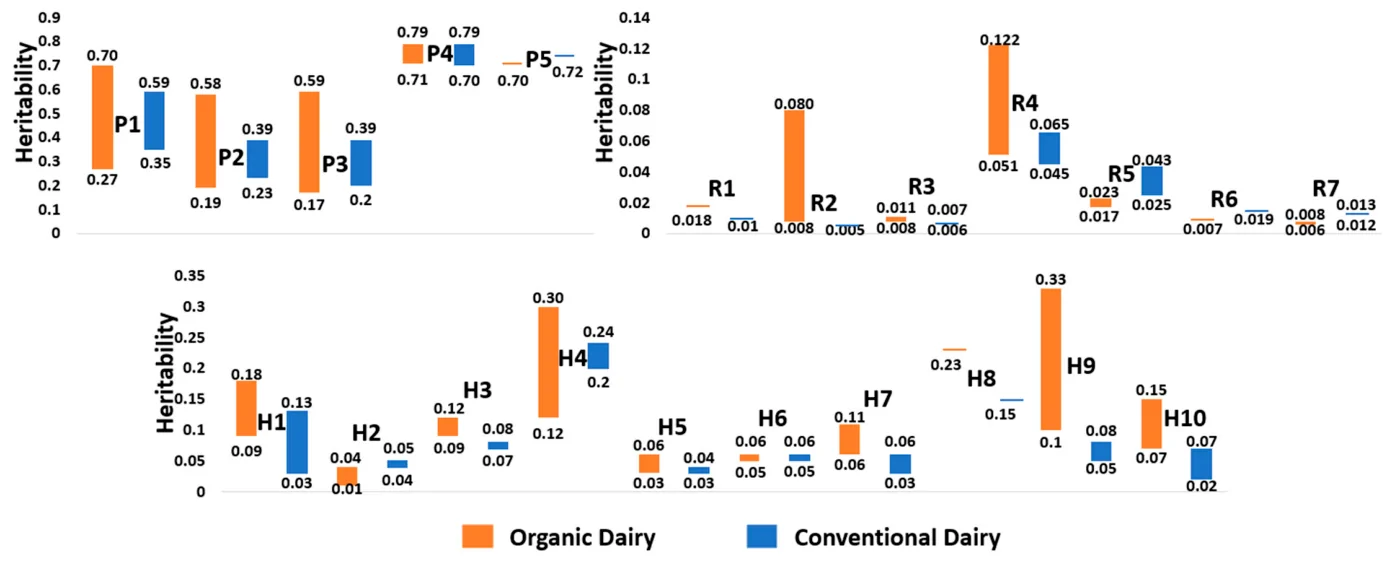
Heritability estimates for productive, reproductive and health/wellbeing traits in organic and conventional dairy systems. P1: milk yield; P2: fat yield; P3: protein yield; P4: fat (%); and P5: protein (%). R1: number of inseminations per conception (heifers); R2: interval from first to last insemination (heifers); R3: non-return rate after first insemination (heifers); R4: interval from calving to first insemination; R5: number of inseminations per conception (cows); R6: interval from first to last insemination (cows) and R7: non-return rate after first insemination (cows). H1: productive life; H2: survival through lactation 1; H3: survival through lactation 2; H4: survival through lactation 3; H5: fertility-determined survival; H6: udder health-determined survival; H7: mastitis; H8: somatic cell score (SCS); H9: digital dermatitis; and H10: ovarian cycle disorders.

Genetic parameters such as heritability, genetic correlations and genomic regions can differ between organic and conventional dairy production systems [41,50,51,52]. The overview of the extent to which genetic variation contributes to phenotypic differences across production systems and trait categories described from 11 published studies between 2006 and 2023 includes 42 traits which grouped into three categories: production (8 traits), health and wellbeing (24 traits), and reproduction (10 traits) (Table S2) [41,50,51,52,53,54,55,56,57,58,59]. However, only traits with heritability records in both organic and conventional dairy systems are demonstrated in Figure 3.

Heritability of milk-production traits ranged from 0.39 to 0.70 under organic management and 0.35 to 0.59 under conventional management, although the magnitude and direction of these differences varied among traits and study populations [41,50,58]. In contrast, milk composition traits were consistently among the most heritable traits regardless of production system, with fat percentage ranging from 0.70 to 0.79 and protein percentage around 0.70–0.72, suggesting that selection for milk quality should be similarly effective in both environments [41,50]. Mature-equivalent 305-days production traits reported only in organic herds showed lower heritabilities (0.12–0.17), indicating a greater influence of environmental factors [53].

Health and wellbeing traits were generally characterized by low to moderate heritabilities, reflecting their complex biological and environmental determinants. However, several traits showed higher heritabilities under organic systems than under conventional systems, including productive life (0.09–0.18 vs. 0.03–0.13), mastitis (0.06–0.11 vs. 0.03–0.06), somatic cell score (0.23 vs. 0.15), and digital dermatitis (0.10–0.33 vs. 0.05–0.08) [41,50,52,55]. Survival traits displayed low heritabilities in both systems, although survival through later lactations (S2 and S3) tended to be more heritable than first-lactation survival [52]. Numerous health traits have only been evaluated in organic populations, including respiratory disease, scours, lameness, metabolic disorders, mortality, health costs, horn fly resistance, and passive immunity indicators, with heritabilities mostly ranging from 0.03 to 0.25, except for white coat coloration, which was moderately heritable (0.44–0.46) [53,54,55,56,57].

Reproductive traits consistently exhibited the lowest heritabilities among the three trait categories, indicating strong environmental influence and slower expected genetic progress. Most fertility measures, such as non-return rate, insemination interval traits, and number of inseminations per conception, had heritabilities below 0.05 in both systems [51]. Nevertheless, some fertility-related traits showed slightly greater genetic variation under organic management, including interval from calving to first insemination (0.051–0.122 vs. 0.045–0.065) and ovarian cycle disorders (0.07–0.15 vs. 0.02–0.07) [41,51]. Conversely, conventional herds tended to show somewhat higher heritabilities for cow fertility traits, such as number of inseminations per conception, interval from first to last insemination, and non-return rate in cows [51].

Overall, the comparison suggests that production traits remain the most heritable and therefore the most responsive to selection in both organic and conventional systems, while health and longevity traits show low to moderate heritability with several traits exhibiting greater genetic variation under organic management. Reproductive traits are generally weakly heritable regardless of system, although specific fertility and reproductive disorder traits may display system-dependent differences. These results indicate that breeding programs for organic production may have particular opportunities to improve health, longevity, and disease-resistance traits while maintaining progress in production performance [41,50,55,58].

### 2.3. Genetic Correlation

**Figure 4:**
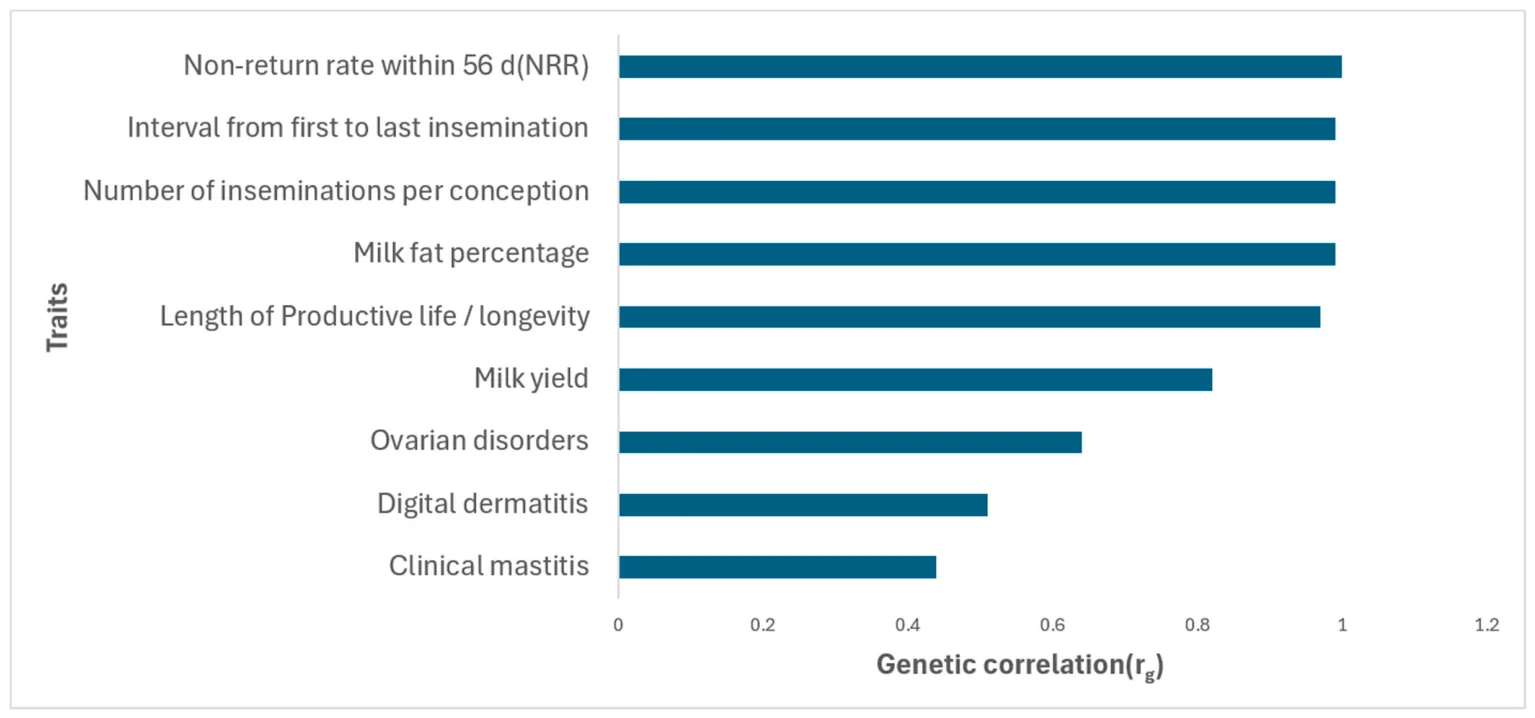
Genetic correlation (r_g_) of different traits between organic and conventionally raised dairy cows.

Genetic correlations between organic and conventional dairy systems indicate that genotype × environment interaction is trait dependent. In Dutch Holsteins, Nauta et al. [50] reported moderate organic–conventional genetic correlations for milk yield and protein yield, with rg values of 0.80 and 0.78, respectively, whereas fat yield showed a higher correlation of 0.88, suggesting weaker G × E for fat yield than for milk or protein production traits (Figure 4). The same study also reported that fat percentage, protein percentage, and somatic cell score had correlations close to unity, indicating limited evidence of G × E for these traits. In contrast, Ahlman et al. [52] found generally high genetic correlations for longevity traits between organic and conventional Swedish dairy systems, ranging from 0.88 to 1.00 in Swedish Holsteins and from 0.80 to 1.00 in Swedish Red cattle, suggesting little biologically important G × E for most survival traits. For Danish Holstein fertility traits, Liu et al. [51] reported wider variation, with rg values ranging from 0.607 for heifer non-return rate to 1.000 for cow non-return rate, indicating stronger G × E for some heifer fertility traits than for cow fertility traits. Shabalina et al. [41] reported low-to-moderate organic–conventional genetic correlations for mastitis, ovarian cycle disorders, and digital dermatitis across pedigree, genomic, and single-step models; however, these estimates came from markedly unequal populations and had limited precision for several low-heritability traits.

### 2.4. Quantitative Trait Loci Mapping

Genome-wide association studies (GWAS) have become more common for finding genomic regions that affect traits like production, health, and longevity in dairy cattle. These studies provide molecular-level proof of genotype by environment interactions (G × E) between organic and conventional production systems. Shabalina et al. [41] did the most thorough GWAS that directly compared organic (1282) and conventional (19,700) cow reference populations. They looked at 40,830 SNPs from conventional and organic German Holstein cows across production, longevity, and health traits. For production traits with moderate to high heritability, milk yield and fat percentage (FP) significantly associated SNPs exhibited extensive overlap between the two populations, with both systems pinpointing the principal genes on BTA14, suggesting that the genetic architecture of these traits remains largely stable across environments. Conversely, for variables with poor heritability related to length of productive life (LPL) and health including mastitis, ovarian cycle disorders (OCD), and digital dermatitis (DD), the significantly correlated SNPs and annotated putative candidate genes varied between the production system. For example, significantly linked SNPs for mastitis in conventional cows were identified on Bos taurus autosomes 6 (*SLC4A4*, *GC*, and *NPFFR2*) and 19 (*FLCN*, *SKAP1*, *ARL4D*, *DHX8*, *TMEM104*) but in the organic group they were found on autosomes 1 (*ALCAM*), 10 (*GLDN*, *CYP19A1*, *USP8*, *GABPB1*, and *HDC*) and 22 (*FAM19A1*). In LPL, candidate SNPs in conventional herds were dispersed throughout the entire genome, with annotations indicating genes such as *DLG1* (immune response) on BTA1, *SEMA5A* (mastitis resistance) on BTA20, *LEP* (metabolic and energy regulation) on BTA4, *NCMAP* and *RCAN3* (preweaning mortality) on BTA2, *CACUL1* (age at first calving) on BTA26, *PDE4D* (fertility-related signaling) on BTA20, and *MTPAP* on BTA13. On the contrary, LPL-related SNPs in organic herds were predominantly located on BTA16 (53.5–64.8 Mb), with potential genes such as *KIAA0040*, *COP1*, *RASAL2*, *RALGPS2*, *BRINP2* (already connected with buffalo fertility), none of which coincided with the conventional LPL loci. In DD, *BNC2* (BTA8), linked to fertility in Brangus heifers, was significant in conventional herds, whereas *ARHGEF12* (BTA15), influencing subclinical mastitis in Norwegian Red cattle, was significant in organic herds. In conventional herds with OCD, candidate genes *AKR1B1* and *AKR1B10* were identified on BTA4 (related to corpus luteum degradation) and *ZNF23* on BTA18 (associated with ovarian dysfunction); organic herds had unique correlations on BTA3 (*PLPPR4*, connected to follicular transformation and ovarian diseases). They also reported that production traits showed more numerous and overlapping significant SNP associations between organic and conventional dairy systems, whereas longevity and health-related functional traits showed fewer and more system-specific SNP associations. This pattern likely reflects the higher heritability, continuous measurement scale, and presence of major-effect loci for production traits, compared with the low heritability, threshold nature, polygenic architecture, environmental sensitivity, and stronger genotype × environment interaction underlying reproductive and health traits.

**Figure 5:**
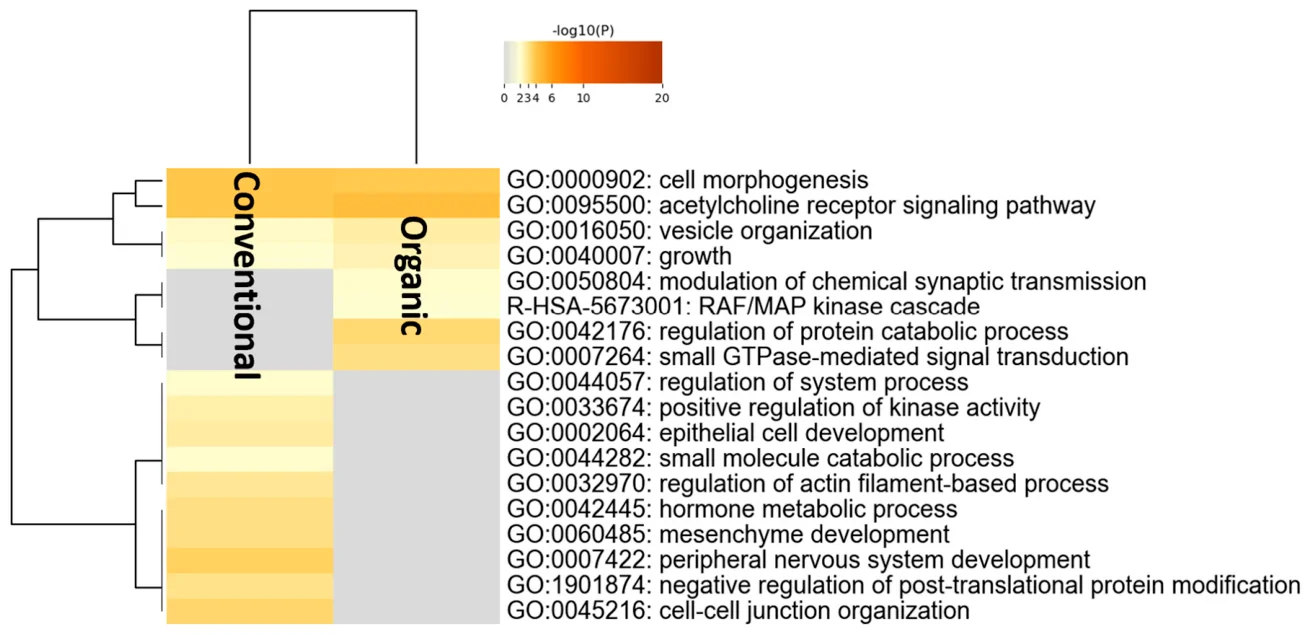
Comparative pathway-enrichment heatmap of organic and conventional dairy associated genes from Shabalina et al. [41] created by Metascape (v3.5.20260701).

Heatmap showing enriched Gene Ontology (GO) and Reactome pathway clusters identified by Metascape using unique gene symbols associated with organic and conventional dairy systems identified by Shabalina et al. [41] (Figure 5). The heatmap indicates that cell morphogenesis and acetylcholine receptor signaling are strongly enriched in both organic and conventional dairy-associated gene lists, suggesting shared involvement of cellular remodeling and neuroendocrine/physiological signaling across production systems. Nonetheless, conventional-associated genes demonstrated greater enrichment for hormone metabolic processes, epithelial cell development, actin filament regulation, small molecule catabolism, and cell–cell junction organization, whereas organic-associated genes demonstrated comparatively greater enrichment for growth, vesicle organization, RAF/MAP kinase signaling, small GTPase-mediated signal transduction, and protein catabolic regulation. These findings imply that while the two systems share fundamental cellular remodeling pathways, the emphasis of these pathways varies: conventional-associated genes favor endocrine-metabolic regulation and epithelial/cytoskeletal organization, whereas organic-associated genes favor adaptive cellular signaling and intracellular trafficking.

Therefore, it is concluded that organic and conventional dairy cattle share similar genetic architecture for highly heritable production traits but differ substantially for low-heritability functional traits such as health, fertility, and longevity. Hence, future breeding programs for organic dairy farming should prioritize genomic selection for disease resistance, robustness, grazing efficiency, and lifetime productivity rather than relying solely on conventional high-yield selection lines.

However, the studies reviewed in this section also have some limitations. Genomic evidence is limited by the narrow geographic and breed base and by unequal reference-population sizes. Most estimates come from Holstein or a few European dairy breeds, while several U.S. studies include organic herds only [41,50,51,52,53,54,55,56,57,58,59]. In the largest direct genomic comparison, 1282 genotyped organic cows were contrasted with 19,700 conventional cows, and only 213 genotyped cows contributed to one genomic longevity correlation; estimates for low-heritability health traits had large standard errors. The GWAS relied on 40,830 SNPs; its system-specific associations, candidate-gene annotations, and downstream pathway enrichments remain exploratory because they have not been independently replicated or functionally validated [41]. Breed, strain, and crossbreeding results are also management- and region-specific, and the economic crossbreeding comparison was simulated rather than prospectively tested [44,45,46,47,48,49]. Accordingly, current results support hypotheses about system-specific selection but are insufficient to define universally applicable organic breeding indices.

## 3. Phenomic Expression Under Divergent Environments: Production, Functional, and Product Quality Traits

### 3.1. Productive Parameters of Organic vs. Conventional Dairy Cows

**Figure 6:**
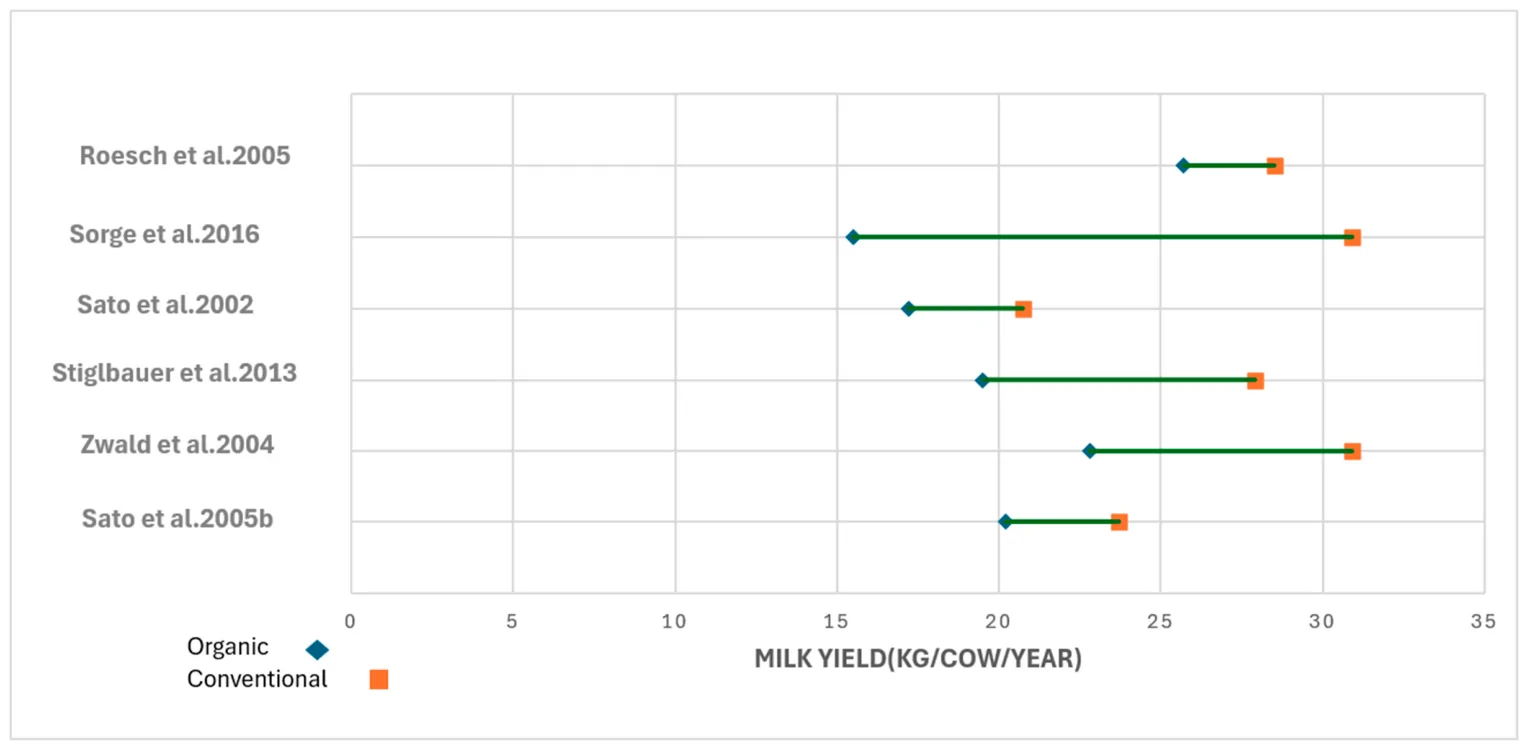
Milk yield (kg/cow/year) of organic vs. conventional dairy cows across different studies [60,61,62,63,64,65].

*Milk production*. The average size of an organic dairy farm in 2008 was 109 cows, and by 2021, it had grown to 142 cows. The proportion of organic farm milk sales jumped from 1.5% in 2008 to 2.3% in 2021. Organic fluid milk sales also increased from 1.6 billion pounds in 2009 to 2.8 billion in 2022, which was a sharp increase from 2.9% to 6.6% during this timeframe (Figure S1) [21]. According to the USDA report, certified organic dairy farms increased from 2004 in 2008 to 3100 in 2019 and then slightly declined to 2478 in 2021, though the overall 23.66% growth was observed during 2008–2021 timeframe. Between 1992 and 2021, the number of organic milk cows in the United States increased tremendously from 2265 to 352,289 cows. A comparative study on Wisconsin organic and conventional dairy by Sato et al. [60] revealed that the organic herds had substantially less animals (ORG = 51.1 vs. CON = 71.1) and mean daily milk yield (ORG = 20.2 kg/day vs. CON = 23.7 kg/day) per cow than the conventional herds (Figure 6). Another study by Zwald et al. [61] found organic farms were smaller (348.2 vs. 173.4) and yielded daily less milk (30.9 kg vs. 22.8 kg) than conventional animals, which is supported by Stiglbauer et al. [62]. A study from 1999 of 69 organic dairy farms in Wisconsin found an average herd size with 61.3 cows and a mean milk yield of 17.2 kg/cow/day, which is below the state average of 20.73 kg/cow/day [63]. Roesch et al. [64] reported that median energy-corrected corrected milk (ECM) yields of 25.7 kg/d for organic cows compared to 28.5 kg/d for integrated production cows at 31 days in milk, with yearly 305-day yields of 5695 kg versus 6059 kg. The findings supported by Sorge et al. [65] who found that organic herds averaging only 15.5 kg/cow per day, in contrast to 30.9 kg for small conventional herds, primarily due to differing breed compositions; organic farms predominantly utilized crossbred cattle, whereas conventional farms primarily employed Holsteins.

A review article encompassing 170 published studies, revealed that organic milk contains 56% more total n-3 polyunsaturated fatty acids, 69% more alpha-linolenic acid (ALA), 57% more combined EPA + DPA + DHA, and 41% more conjugated linoleic acid (CLA) compared to conventional milk, with the n-6:n-3 ratio being 71% lower and the LA:ALA ratio 93% lower in organic milk [66]. Conventional milk compared to organic milk, 2.4 times higher for LA/ALA, 2.5 times higher for v-6/v-3, and 2.0 times higher for v-6/(v-3 + CLA). Organic milk has substantially greater levels of total v-3, ALA, and CLA than conventional milk (62%, 60%, and 18%, respectively) [39]. They agreed with the previous findings [67,68] that, compared to cows without regular access to pasture and fed substantial amounts of forages, milk from these forage-eating cows has higher concentrations of ALA, CLA, and the long-chain omega-3s EPA and DPA, and lower levels of LA and other omega-6 FAs. These results support a strong contribution of grass- and forage-based feeding to milk fatty-acid composition, rather than an effect of certification alone; whether the observed compositional differences produce clinically meaningful benefits for children, pregnant women, or people at cardiovascular risk has not been established [39,66,67,68,69]. In a study conducted across 67 UK farms, Ormston et al. [43] found that while conventional milk had higher concentrations of total protein, casein, lactose, and monounsaturated fatty acid (MUFA), organic milk had significantly higher concentrations of ALA, rumenic acid, EPA, and total polyunsaturated fatty acid (PUFA). Additionally, conventional farms were more efficient at converting overall diet dry matter into milk, fat, and protein, whereas organic farms demonstrated superior efficiency in converting conserved forages and concentrates in particular. Nogalski et al. [69] similarly reported a daily milk yield that was 11.4 kg lower than conventional milk (16.7 vs. 28.1 kg), characterized by a more favorable fatty acid profile, including elevated levels of PUFA, n-3 PUFA, trans-vaccenic acid, linolenic acid, CLA, and a higher desaturase index, whereas conventional milk exhibited greater concentrations of total fat, protein, and lactose. Manuelian et al. [70] discovered that organic and conventional Holstein–Friesian milk exhibited considerable similarity in most compositional parameters, with only a propensity for higher levels of PUFA and n-3 FA in organic milk and lower levels of protein and casein.

A year-long Danish study discovered that organically produced milk had significantly higher molybdenum but lower barium, europium, manganese, and zinc than conventional milk. However, the observed difference is due to the feeding and seasonal effects over the production system as a whole [71]. According to Stergiadis et al. [72], the main factor influencing variations in mineral content in both conventional and organic farms was grazing intensity rather than certification status alone.

In addition to fatty-acid composition, production-related residues are relevant to exposure assessment; however, retail surveys do not by themselves establish differences in clinical health outcomes. Welsh et al. [73] examined retail milk samples from nine regions in the United States and discovered that current-use pesticides (5 of 15 tested) and antibiotics (5 of 13 tested) were present in conventional milk but absent in organic milk. Furthermore, multiple conventional samples exceeded federal limits for sulfamethazine (37%) and sulfathiazole (26%). Additionally, concentrations of bovine growth hormone and IGF-1 in conventional milk were 20 and 3 times higher, respectively, than in organic samples. Within this retail sample, the findings were consistent with lower occurrence of the measured production-related contaminants in certified organic milk, but one survey cannot establish complete elimination, population exposure, or a resulting health advantage [73]. A study by Cicconi-Hogan et al. [74] stated that *Staphylococcus aureus* is found predominantly in bulk tank milk of organic farms (62%) when compared to conventional non-grazing (42%) and conventional grazing (43%), though organic farms have lower coliform contamination than conventional farms. Older housing facilities, fewer employees for mastitis treatment, and more cows with teat injury were frequently associated with *S. aureus* incidence, which may have contributed to worse udder health and milk hygiene. Overall, the feeding system, housing conditions, and disease management techniques all had a significant impact on milk quality in both systems. Another study by this research team also mentioned that organic and traditional grazing systems of 292 farms in Oregon, Wisconsin, and New York did not significantly differ in bulk-tank somatic cell count (SCC) [75].

### 3.2. Meat

The production system can have a substantial impact on meat quality in terms of carcass composition, fatty acid profile, and sensory attributes between conventional and organic dairy cattle systems. A study on direct comparison of organic concentrate fed (ORG), organic grass fed (GRS) with conventional (CONV) dairy beef steer’s meat quality by Bjorklund et al. [76] found that the oleic acid (C18:1) content of the ORG steers’ fat (47.1%) was higher than that of the GRS (36.1%) and CONV steers (39.9%). Furthermore, the ORG (42.1%) and CONV (40.4%) steers had higher levels of monounsaturated fat than the GRS steers (21.9%). Additionally, the ORG (12.9%) and CONV (10.0%) steers exhibited higher n-6-to-n-3 fat ratios than the GRS (1.4%) steers. Additionally, Phillips et al. [77] showed that compared to purebred Holstein steers, crossbred dairy steers produced beef with higher omega-3 fatty acid contents and a lower omega-6/3 ratio, as well as more preference to crossbred steak, indicating that breed selection within organic systems can further enhance the nutritional profile of beef. However, these nutritional benefits of grass-fed beef came at the expense of less palatability. When compared to conventionally finished or partially grain-supplemented organic beef, consumer sensory panels consistently gave grass-fed beef lower ratings for overall liking, flavor, texture, and juiciness while giving it higher ratings for toughness and off-flavor [76]. Higher-energy grain feeding in conventional systems may result in greater sized carcasses and increased marbling, which can improve juiciness and tenderness. Though, in terms of sensory attributes, ORG and CONV did not show any difference, rather than ORG (73.3) demonstrated a higher taste preference compared to the GRS (56.8) and CONV (69.2) steers [76]. The above evidence suggested that a mixed forage-concentrate system can provide an acceptable balance between consumer acceptance and nutritional quality for the production of organic dairy beef. Overall, the small number of comparisons suggests that feeding regimen and breed can alter carcass traits, fatty-acid composition, and sensory quality; describing organic beef as healthier is not warranted because the observed compositional differences have not been linked to clinical consumer outcomes [76,77].

### 3.3. Feeds and Feeding

A key distinction between organic and conventional dairy systems is feeding management, especially as organic production depends more on pasture-based diets and certified organic feed ingredients. Dry matter intake, nutritional digestibility, feed efficiency, and milk output and composition are all directly impacted by the feed’s composition and quality. Orjales et al. [78] stated that dry matter intake (DMI) was approximately 25% lower in organically managed cows (17.0 kg DMI/day) than conventional non-grazing cows (25.4 kg DMI/day), mostly due to a reduced provision of concentrated feed in the diet of organic cows. The rations on organic farms had a markedly higher proportion of acid detergent fiber (ADF) (27.4 vs. 21.4) and a lower organic matter (OM) (92.3 vs. 93.3) content compared to conventional farming systems, which make organic diets less digestible. Moreover, the energy density of forage in organic systems is significantly lower than in conventional systems, primarily due to the restricted utilization of corn silage and an increased dependence on hay, with the scarcity or minimal inclusion of energy feeds also resulting from their elevated costs and limited availability of certified organic ingredients. They also mentioned that a lower amount of total digestible protein reached the gut on organic cows due to the energy and protein imbalance in organic diets. Compared to their conventional counterparts, organic Jersey cows had reduced feed efficiency (−16%) and milk nitrogen efficiency (−15.5%), which may have been caused by an average 10.4% increase in dry matter consumption [79]. In organic dairy systems, reducing the size of the forage particles greatly improved the apparent total-tract digestibility of nutrients, and energy-corrected milk output increased (27.0 vs. 29.3 kg/d) without changing feed efficiency or milk solids [80].

In one organic feeding trial, cows fed liquid molasses had lower milk and plasma urea-nitrogen concentrations and improved nitrogen utilization, but higher total dry matter intake and no change in milk yield or milk components, compared with cows fed corn meal [81]. Ghedini et al. [82] found that replacing corn meals with increasing levels of liquid molasses results in a linear reduction of dry matter intake, milk yield, and lactose concentration. Though the milk fat and milk protein were not affected, the enterolactone concentration changed cubically. Overall, liquid molasses substitution with corn meal adversely affected the cow performance. In both summer and spring season, short term grazing trial on annual forage crops (AFC) did not show any difference in dry matter intake, milk yield, or ruminal fermentation and no consistence increase of herbage biomass production in jersey cows compared with traditional legume-grass pasture [83]. Overall, AFC inclusion did not show any detrimental effect on performance. In comparison with the non-grazing season, the grazing season showed the greatest total conjugated linoleic acid (CLA) concentration in milk. Flaxseed supplementation during winter results total CLA concentrations raised by 9.0%, boosted omega-3 fatty acid concentrations by 88%, and reduced total milk SFA concentrations by 3.1 percentage points [84]. Arndt et al. [85] found that seaweed-supplemented cow’s manure decreased carbon without impacting methane and nitrous oxide emissions. The total amount of carbon and nitrogen mostly went down, except in the 3% seaweed supplementation group, where carbon and nitrogen went up. Seaweed supplements are not harmful and may lessen the effects of cow manure on the environment. Among the survey respondent farmers, all of them know seaweed (Chondrus crispus or *C. crispus*) as feed and 34% were using seaweed at that time. Supplementation of *C. crispus* at 6% of the diet DM reduced CH4 generation by 13.9% without affecting milk quantity or composition. Overall, *C. crispus* may have an impact on methane mitigation strategy [86].

Overall, conventional systems predominantly utilize energy-dense concentrates and total mixed rations to optimize dry matter intake and milk production, whereas organic systems prioritize forage- and pasture-based feeding. In the cited studies, forage-rich diets were associated with higher concentrations of selected fatty acids but also with lower digestibility or feed efficiency in some settings [43,78,79]. These are primarily feeding-system effects and should not be attributed to certification independently of diet composition, forage quality, and concentrate allowance.

### 3.4. Reproductive Performances

**Figure 7:**
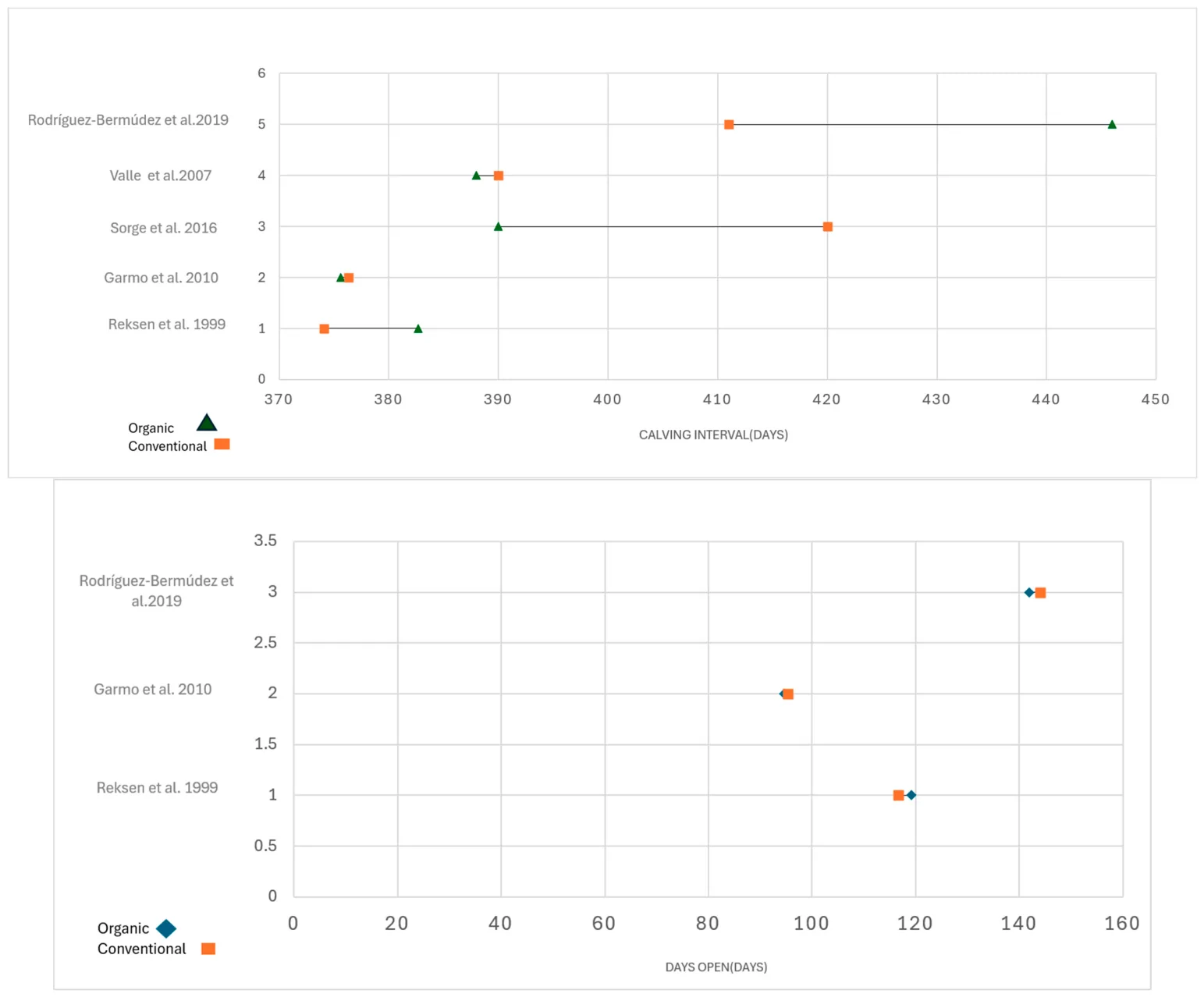

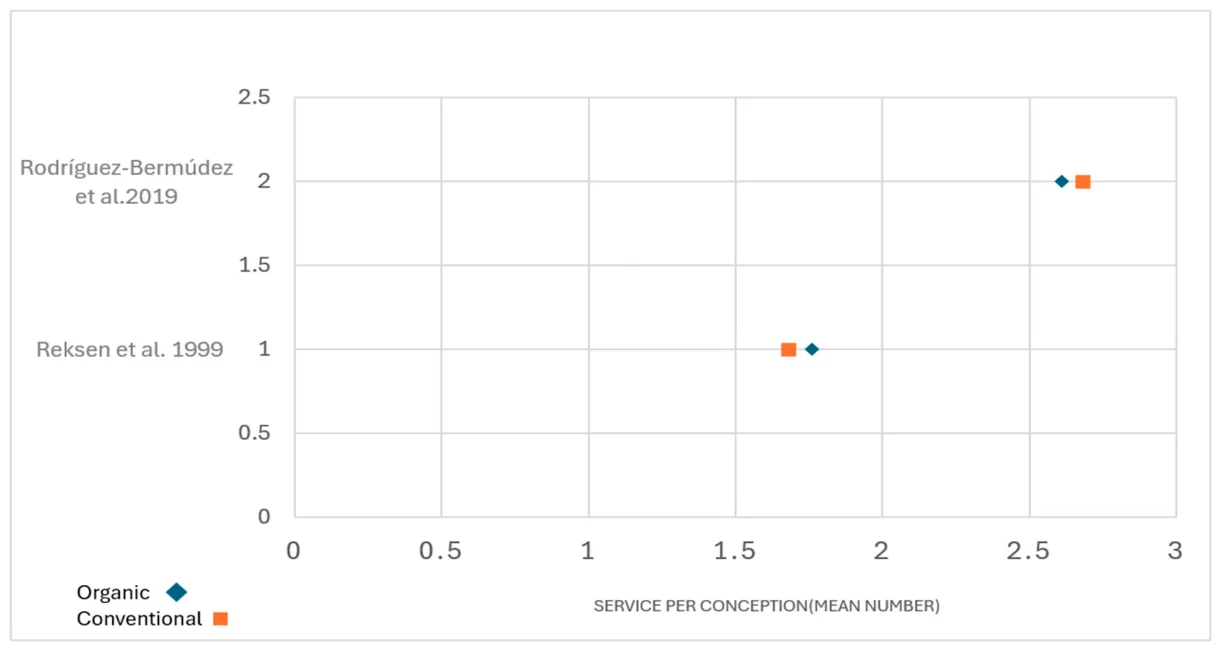
Reproductive parameters {calving interval (days), days open (days), service per conception} of organic vs. conventional dairy cows across different studies [48,65,87,88,89].

Comparative analyses of reproductive performance in organic vs. conventional dairy systems have produced conflicting results, mostly because of variations in management approaches, breed composition, geographic factors, and the restriction on hormonal synchronization protocols in organic production. Though the milk yield is consistently less in organic farms than conventional [64,65], in general, the reproductive performance of organic farms was either somewhat better than or equal to that of conventional farms. The foundational cohort research conducted by Reksen et al. [87] utilizing Norwegian herd tracking data indicated that no consistent differences were observed in calving interval, the time from calving to first and last artificial insemination, or artificial inseminations per cow between organic and conventional system; nevertheless, cows under organic system exhibited considerably fewer days open. The Swiss study found no significant differences between organic and conventional farms in days to first service, services per conception, days open, days from first service and conception and calving interval [64]. Similarly, the 2019 Spanish study by Rodríguez-Bermúdez et al. [48] stated that no statistically significant differences were detected among farming systems for calving interval, age at first service, age at first conception, or age at first calving, though organically grown Holstein–Friesian cows had shorter calving-to-first-service and calving-to-conception intervals compared to pasture-based conventional cows (Figure 7). No disparities were seen in the interval to first artificial insemination, interval to last artificial insemination, or calving interval between organic and conventional cows [88,89]. Nauta et al. [50] reported that longer calving interval (approximately 403 days) in Dutch organic farms that had converted from conventional systems, as well as the age at first calving (AFC) of these organic farms rose from 26 to 27 months, but the AFC for conventional farms remained at 26 months. A study by Sorge et al. [65] observed that reduced incidences of dystocia and stillbirths, with heifers and cows requiring assistance during calving on organic farms in contrast to small conventional herds. Additionally, the reported age at first calving was greater in organic herds (27 months) compared to most conventional operations (24–25). A Swedish study indicated that organic cows had shorter calving intervals and reduced durations from calving to first and last artificial insemination compared to conventional cows [90]. Manríquez et al. [91] found that the incidence risks of reproductive problems during lactation, such as retained fetal membranes, metritis, endometritis, and pyometra, were comparable across big certified organic dairies and conventionally managed herds. More recently, Shrestha et al. [92] emphasized that although reduced conception rate due to the restricted use of timed-AI synchronization programs in certified organic systems in comparison to conventional herds, organic cows have longer productive lifespans and significantly lower annual culling rates (roughly 20–21% vs. 35–38%).

Collectively, these findings indicate that while organic systems encounter intrinsic reproductive management limitations, the prevalence of reproductive diseases and overall fertility outcomes may not be inferior to those found in conventional dairy production systems. Though these differences cannot be assigned to certification alone because milk yield, breed composition, voluntary waiting period, culling policy, pasture exposure, and restrictions on hormonal synchronization differed among studies [64,65,87,88,89,90,91,92].

Despite providing valuable insights, the studies discussed in this section had several limitations. For example, most farm-level phenomic comparisons are observational; farms self-select into organic or conventional management and simultaneously differ in breed, herd size, yield, pasture exposure, diet, housing, season, and veterinary practices, preventing causal attribution to certification alone [43,60,61,62,63,64,65,69,70,71,72,73,74,75,78,79,87,88,89,90,91]. Studies also use different milk-yield expressions, sampling schedules, lactation stages, reproductive definitions, and recording periods, which limit quantitative comparison across countries and years. Evidence on dairy-beef quality and alternative feeds comes from few herds and often small or short-term trials; with limited external validation [76,77,80,81,82,83,84,85,86]. Fertility comparisons are further confounded by milk yield, breed composition, voluntary waiting period, culling policy, and restrictions on hormonal synchronization [64,65,87,88,89,90,91,92]. Thus, the direction of individual differences cannot be generalized to populations other than the ones studied.

## 4. Health Outcomes Under Contrasting Systems: Animal Health, Antimicrobial Resistance, and Human Implications

In organic dairy farming, restricted use of synthetic drugs and antibiotics encourages preventive herd health management. However, this can make it harder to deal with diseases like mastitis, lameness, metabolic disorders, and reproductive infections when instant treatment is needed. In comparison to conventional grazing (CON-GR) and nongrazing (CON-NG) farms, organic farmers reported fewer cases of ketosis and calf diarrhea, displaced abomasum, metritis, and pneumonia (in both cows and calves) over the 120-day observation period [93].

### 4.1. Mastitis

A study by Ruegg [94] found, the prevalence of mastitis and bulk tank somatic cell counts (SCCs) were similar between organic and conventional farms, though they used non-antibiotic therapies, such as herbal treatments instead of intramammary antibiotic as like conventional dairy. Ruegg also observes that organic farms frequently lack established methods for mastitis diagnosis and treatment and veterinary consultation compared to conventional farms. This study highlights that treatment tactics are diverse in two systems, where organic systems prioritize prevention and conventional systems depending more on curative measures. Another study by Pol and Ruegg [95], found a greater prevalence of contagious pathogens (5.4% *Staphylococcus aureus* and 2.3% *Streptococcus agalactiae*) from individual quarter milk samples of organic cows in contrast to samples from cows on conventional farms (2.9% *Staph. aureus* and 0.8% *Strep. agalactiae*). This is due to inability to administer long-acting intramammary antibiotics during dry-off. Zwald et al. [61] found SCC levels in a greater proportion of ORG cattle compared to CON herds, though Pol and Ruegg [96] and Sato et al. [97] did not find any discrepancies in bulk milk bacterial counts in both systems. Sato et al., 2005 [97] also did not find any significant difference in clinical mastitis occurrence and yearly culling rate between farm types. Hamilton et al. [98] and Pol and Ruegg [95] found approximately 8 to 9% culling observed in both organic and conventional herds, owing to mastitis rather not related to the herd system. Pol and Ruegg [96] found number of clinical mastitis reported cases per year, respiratory disease, metritis and treated clinical mastitis cases per year was significantly higher (*p* < 0.001) in conventional than organic cows, whereas reported foot infection cases per year were higher in organic cows. Around 79.8% of conventional farms use antibiotics to treat mastitis compared to 0% of organic farms. Another study in Wisconsin found, lower culling rate (25%) than conventional farms though the bulk tank somatic cell count was 329,000 cells/mL, and the mastitis incidence was 42 instances per 100 cow-years in these organic herds, which were equivalent to conventional herds [63].

### 4.2. Parasitic Diseases

A cross-sectional study by Sorge et al. [99] assessed parasite management among 33 organic (ORG), 15 small conventional (SC), and 13 medium-sized conventional (MC) dairy herds in Minnesota and found that ORG herds exhibited significantly elevated fecal egg counts (FEC) of strongyle-type gastrointestinal parasites (ORG: 6.6 ± 2.1 eggs/g vs. SC: 0.5 ± 0.3, MC: 0.8 ± 0.7; *p* < 0.01). This could be a result of greater pasture access for organic cows, where infective larvae are predominantly present. Numerically, Trichuris was found more in conventional farms than in organic farms. In the case of ectoparasite infestation, like head and tail mange characterized by frequent scratching and rubbing, hair loss, and scabby skin lesions, the results did not differ between organic and conventional farm types. However, a significant difference was observed for treating this ectoparasite infestation, such as conventional farms using commercial parasiticides where organic farms use iodine spray, brown sugar, and diatomaceous earth.

For fly control, sticky tapes are used by a significantly higher (*p* < 0.01) number of organic farms than conventional farms. Though the commonly used fly control measure is pour-on solutions, spraying or fogging of barns and fly sprays, organic farms exclusively use walkway traps, parasitic wasps, and baited jugs to control the flies. Sato et al. [97] found significantly greater burden of Ostertagia ostertagi antigen from an ELISA assay in organic farms compared to conventional farms during both seasons (March and September) in paired t-tests while these differences did not sustain when they did a mixed linear model considering for season, grazing intensity, and milk production. Richert et al. [93] stated that about half as many farmers on organic farms reported intestinal parasites as an issue or regularly applied prophylactic treatments as compared to conventional grazing and non-grazing farms. In contrast, a study on 36 organic farms of Northern California found that over half of the organic dairy producers did not consider external pests and parasites to be a major problem on their farms [100]. The disappearance of the Ostertagia contrast after adjustment illustrates how an apparent organic–conventional difference can be explained by grazing intensity, season, and production level rather than certification itself.

### 4.3. Lameness

Conventional nongrazing herds tended to have higher farmer-reported lameness rates than conventional grazing and organic herds; reported rates ranged from 0 to 0.89 cases per cow-year (*p* = 0.071) [101]. Farms that regularly used footbaths also had higher lameness prevalence, but this cross-sectional association may reflect greater footbath use on farms already experiencing lameness rather than a harmful effect of footbaths.

### 4.4. Bacterial Prevalence and Antimicrobial Resistance

In a one-year longitudinal study of 129 farms in Minnesota, Wisconsin, Michigan, and New York, *Salmonella* was isolated from 4.9% of bovine fecal samples. Open housing, unenclosed feed storage, no monensin use in calves, surface-water access, and grazing on recently spread liquid manure were associated with higher *Salmonella* shedding [102]. This team also found that the incidence of *Salmonella* spp. on conventional and organic farms did not differ significantly [103]. Ray et al. [104] reported similar susceptibility for most antimicrobial agents, although isolates from conventional farms had greater streptomycin resistance. Conventional farms were more likely to yield multidrug-resistant Salmonella, but the difference was not statistically significant.

Among culled cows, conventional animals had higher levels of tetracycline-resistant *Escherichia coli*, third-generation cephalosporin-resistant *Escherichia coli*, and the erm (B) resistance gene in colon contents than organic animals. However, antimicrobial resistance detected on carcasses and meat trimmings did not differ between production systems [105].

Overall resistance of Campylobacter isolates to eight antimicrobials did not differ significantly by farm type. However, tetracycline resistance was higher in conventional than organic isolates (58% vs. 48%; *p* < 0.01), was associated with the tetO gene, and a greater proportion of organic isolates were highly susceptible (MIC <= 0.25 micrograms/mL) [106].

A comparison of 30 organic and 30 conventional Wisconsin dairy farms found no significant difference in Campylobacter prevalence in cow feces. Forty-five percent of isolates were tetracycline resistant, whereas all isolates were susceptible to gentamicin and erythromycin. The authors concluded that resistance to ciprofloxacin, gentamicin, erythromycin, and tetracycline was not associated with antimicrobial-use restrictions when Campylobacter was used as an indicator organism [107]. In a separate study, Sato et al. [60] found higher resistance to ampicillin, streptomycin, kanamycin, gentamicin, chloramphenicol, tetracycline, and sulfamethoxazole among Escherichia coli isolates from conventional than organic farms.

Testing of Shiga toxin-producing Escherichia coli showed that all isolates from Minnesota organic farms were susceptible to chloramphenicol, gentamicin, and trimethoprim/sulfamethoxazole, whereas 3 of 29 conventional-farm isolates were resistant to one or more of these agents. Spectinomycin resistance was higher in conventional than organic isolates (72.4% vs. 39.1%) [108].

A survey of New York dairy farmers found that organic respondents expressed greater concern about antimicrobial resistance and its implications for human health, whereas conventional respondents more often considered their current antimicrobial use prudent and were concerned that further restrictions could compromise cattle health. Both groups prioritized disease prevention through herd-health management [109]. These studies differed in organisms, sample matrices, antimicrobial panels, geography, and sampling design, so they do not support a single system-wide conclusion about antimicrobial resistance.

### 4.5. Potential Human Health Implications

The consumption of organic foods has been linked to several potential health benefits for humans; however, conclusive causation evidence is still scarce. Vigar et al. [110] conducted a systematic review of 35 human studies, revealing that increased organic food consumption in observational cohorts was associated with a decreased incidence of infertility, birth defects, allergic sensitization, otitis media, pre-eclampsia, metabolic syndrome, elevated BMI, and non-Hodgkin lymphoma, whereas randomized clinical trials were predominantly short-term and less definitive. Organic diets may provide advantages, as organically grown crops contain higher antioxidant and lower amounts of pesticide residues as well as certain heavy metals, such as cadmium, in comparison to conventional meals [111]. A dietary intervention study on thirteen Australian adults for substituting conventional foods with organic foods for seven days revealed that diminished urinary pesticide (organophosphate) metabolite excretion, in certain instances by as much as 90%, signifying swift decreases in dietary pesticide exposure [112]. In the French NutriNet-Santé study, people who ate more organic foods were less likely to be overweight or obese. The risk of obesity dropped by 37% during follow-up among the people who ate the most organic foods [113]. A greater consumption of organic food correlated with a diminished total cancer risk (HR = 0.75; 95% CI: 0.63–0.88), especially regarding non-Hodgkin lymphoma [114]. A review study by Wittwer et al. [111] stated that polyunsaturated fatty acids, which are abundant in organic dairy products may benefit the gut microbiota and intestinal epithelium. Organic dairy products’ higher ω-3:ω-6 ratio may reduce inflammation and gut dysbiosis and higher levels of the fat-soluble vitamins A, D, and E could have anti-inflammatory effects on the gut and possibly improve the makeup of the microbiome. Finally, organic management practices that do not allow xenobiotics may lower the danger of intestinal dysbiosis caused by pesticides, antibiotics, or heavy metals.

Nevertheless, the evidence summarized in this section also had potential drawbacks. Disease comparisons frequently rely on cross-sectional visits, farmer questionnaires, or treatment records; differences in diagnostic intensity, record keeping, antibiotic eligibility, and veterinary involvement can create under-ascertainment or reporting bias [61,63,93,94,95,96,97,98,99,100,101]. Antimicrobial-resistance studies varied in bacterial species, sample matrices, antimicrobial panels, interpretive criteria, sampling years, and numbers of resistant isolates, so their results establish prevalence associations rather than causal effects of certification or transmission risk to people [60,102,103,104,105,106,107,108]. Human-health evidence largely concerns overall organic-food consumption, not organic dairy specifically [110,113,114]. The 7-day intervention included only 13 adults and measured urinary pesticide metabolites rather than clinical outcomes [112], whereas proposed gut-microbiome benefits are largely mechanistic and untested in long-term controlled trials [111]. Thus, no conclusive causal advantage to human health from organic dairy can be shown.

## 5. System-Level Consequences: Environmental Sustainability, Welfare, and Economic Trade-Offs

### 5.1. Animal Welfare

The health and welfare of organic dairy cattle differ from conventional systems due to antibiotic restrictions and the possible welfare benefits from the use of obligatory grazing time. Alvåsen et al. [115] discovered that organically managed dairy herds in Sweden, among 4252 herds, exhibited significantly lower on-farm mortality rates compared to conventional herds, even after accounting for herd size, breed, and milk yield. This finding substantiates that the organic production environment provides a quantifiable survival advantage at the herd level. A study by Bergman et al. [116], compared the animal welfare parameters such as calf management including disinfecting navels, weaning age, dehorning age, dehorning method, pain relief methods during dehorning, calving area, time of first colostrum, colostrum intake, frequency of milk fed to pre-weaned calves and for the adult cows; udder hygiene, hock lesion, lameness, body condition score, written health records, treatment records, regular use of veterinarian, personnel training by veterinarian, protocols developed by veterinarian, protocol for mastitis, and milking routine among organic (ORG), conventional grazing (CON-GR) and non-grazing (CON-NG) dairy herds. They found ORG farms had the significantly lowest prevalence of hock lesions and superior leg condition scores than both CON-NG, CON-GR systems. Calves on ORG farms were weaned at a later age (11.6 weeks) and received greater milk quantities (5.5 L/day), but calves on CON-GR and CON-NG farms were weaned sooner (8.0–8.3 weeks) and fed less (4.8 L/day). A greater proportion of organic herds passed the welfare criterion of keeping cows with BCS ≤ 4.5 than conventional grazing and nongrazing herds, indicating fewer excessively overconditioned cows rather than a higher BCS in the organic herds. Conventional non-grazing herds were more inclined to adhere to veterinarian-developed standards and perform routine veterinary visits. The data indicate that organic dairy farms may promote enhanced well-being outcomes for calves and cows; nevertheless, variations in veterinarian management and adherence to protocols reveal opportunities for improvement across all systems.

### 5.2. Environmental Health

The environmental impacts of organic and conventional dairy production have been analyzed from various perspectives, including greenhouse gas emissions, nitrogen dynamics, soil erosion, and soil health. A recent meta-analysis indicated that organic farming yields a significant reduction in soil loss, with a median decrease of 26%, and in terms of overall soil fertility, increased earthworm biomass and abundance were observed by 78% and 94%, respectively [117]. However, De Boer [118] described that organic systems can naturally result in higher methane emissions per unit of milk production due to lower milk yield and high forage diets compared with conventional cows. Organic farming, which restricts synthetic inputs and emphasizes soil enhancement, offers distinct advantages for soil conservation, water protection, and climate adaptation. Thus, organic farming may help address contemporary environmental and resource issues and is rightly regarded as a fundamental strategy for sustainable land use. Skinner et al. [119] found that long-term organic farming decreases nitrous oxide (N_2_O) emissions by 40.2% per hectare in comparison to conventional systems. They also concluded that an effective strategy for reducing greenhouse gas emissions in the agricultural industry is the use of organic farming practices. Organic systems utilized 92% fewer pesticides and 76% less mineral nitrogen compared to conventional systems. Organic farming systems, particularly compost-based biodynamic methods, demonstrated improved soil health, increased diversity of micro- and macro-fauna, and a variety of weed species. On the contrary, the conventional system showed increased N_2_O emissions and less efficiency of nitrogen utilization [120]. Over the long term, the organic farming system proved to be more efficient than the conventional farming system in mitigating soil erosion and, consequently, in preserving soil productivity [121,122].

### 5.3. Economic Viability

The comparative aspect of profitability between organic and conventional dairy farming continuously indicates a complex trade-off between increased production costs and greater milk price premiums in organic systems. A comparative study by O’Hara and Parsons [123] was conducted in Minnesota and Vermont to find out the economic impacts of organic dairy farms compared to similar-sized conventional dairy farms. They found that compared to conventional dairy, Vermont’s organic dairy revenue results in 46% more jobs, 39% additional labor income, 33% greater gross state product, and 3% greater overall economic output and organic farms in Minnesota generate 4% more output, 9% more labor income, 11% more gross state product, and 12% more jobs. These findings summarize that, in comparison to conventional systems, organic dairy offers greater economic benefits at the community level in addition to farm profitability in Northeast and Upper Midwest regions of the USA.

There was no significant difference in dry-off (intramammary and systemic) treatment cost between conventional ($13.30) and organic farms ($7.43). However, the estimated cost for treating clinical mastitis was over twice as high for conventional farms ($28.48) compared to organic farms ($11.33; *p* = 0.02) [94]. Compared to similarly sized conventional dairy farms, certified organic dairy farms typically received higher prices for milk and faced increased feed costs per 100 pounds of milk sold which showed higher returns over operating costs and total economic costs in 2016 and 2021 [21]. Nehring et al. [124] used a stochastic production frontier model to show that organic farms with 75 or more cows made greater net return than conventional farms of the same size because of the higher price of organic milk. Similar to organic milk, organically produced meat (beef) has a premium price that leads to 43% higher profit compared to conventional steers [125].

**Figure 8:**
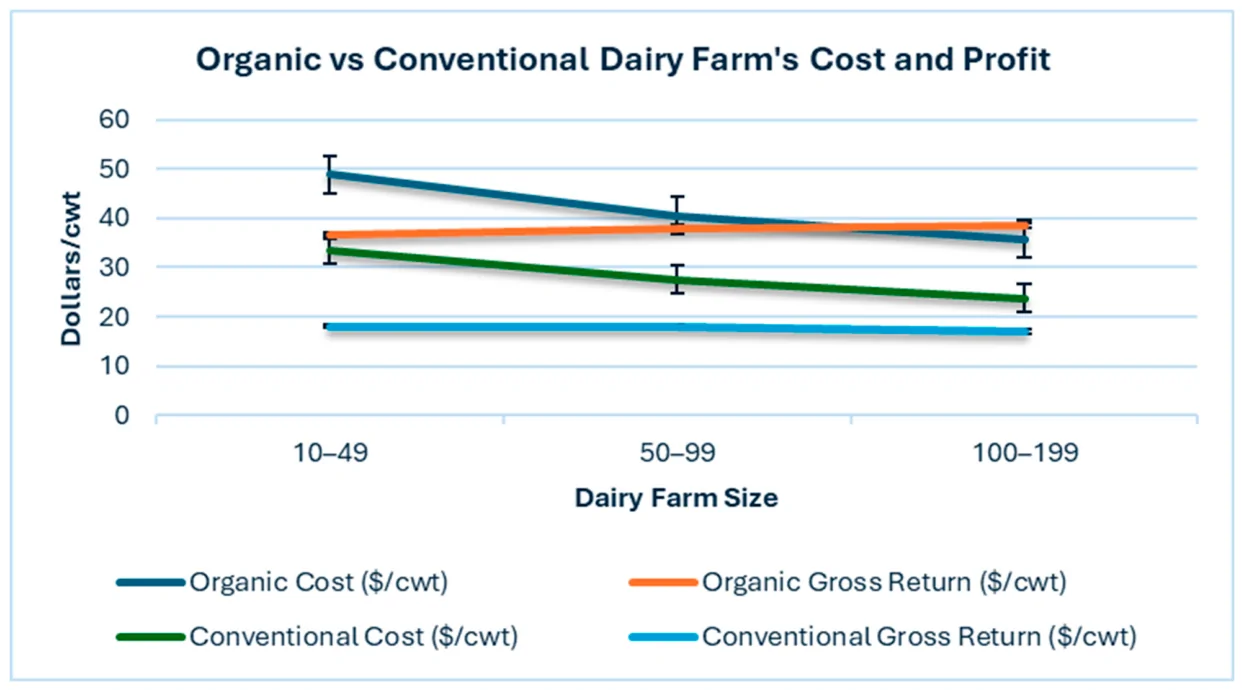
Profitability of organic and conventional dairy farms based on their farm size [126,127].

According to the USDA survey, organic dairy farms had much higher production expenses than conventional farms across all size categories in 2016. Nonetheless, they also attained significantly elevated gross returns, particularly within the 100–199 cow range, where organic farms realized positive net returns ($2.45/cwt) in contrast to the negative returns ($5.29/cwt) observed in conventional farms. Smaller conventional farms (10–99 cows) routinely incurred net losses, but comparably sized organic farms performed marginally better albeit still operating at a deficit. However, organic farms with 50–99 cows approached break even at −$2.52/cwt, while conventional farms faced negative net return of $9.24/cwt (Figure 8) [126,127].

Overall, the above-mentioned findings indicate that organic dairy farming can achieve greater financial output, especially for mid-to-large-scale operations, despite incurring higher input costs and experiencing increased market volatility due to the premium price of the products and lower per-cow variable costs. However, regional market conditions, herd management practices, and the stability of organic milk prices significantly influence profitability.

However, welfare comparisons are primarily herd-level or cross-sectional assessments of a few indicators and cannot fully separate organic certification from herd size, breed, grazing, farmer decisions, veterinary involvement, or recording quality [115,116]. Economic findings are specific to particular U.S. regions, survey years, and modeling assumptions and remain highly dependent on organic milk premiums, feed and labor costs, herd size, policy support, and market volatility [21,123,124,125,126,127]. No cited study measured welfare, environmental, and economic outcomes simultaneously on the same farms over time, leaving integrated system-level trade-offs uncertain.

## 6. Conclusions

This analysis highlights major regulatory and management differences between organic and conventional dairy production, and suggests that biological benefits of each system are context-specific, and should be considered in light of the limits of the existing information. Most of the organic-conventional comparative studies were observational, geographically concentrated in Europe or North America, and conducted within particular seasons, farm networks, or market periods. Farms often differed simultaneously in pasture access, grazing intensity, diet, breed composition, herd size, housing, veterinary protocols, farmer objectives, and market conditions. Genomic studies suggest substantial sharing of architecture for production traits and possible G × E for selected functional traits, but the principal GWAS comparison used markedly unequal organic and conventional populations (1282 vs. 19,700 cows), and the system-specific signals lack independent replication. Conventional herds generally achieved higher milk yield by high feed intake and selective breeding, while the pasture and forage-rich diet of organic production were associated with higher concentrations of some n-3 fatty acids and conjugated linoleic acid. These compositional differences do not demonstrate that organic milk is healthier, and human-health evidence remains largely indirect, observational, and focused on total organic diets rather than dairy. Some organic cohorts showed favorable mortality, hock-lesion, or leg-condition outcomes, but welfare findings varied by indicator and were confounded by grazing, housing, herd size, breed, veterinary involvement, and treatment practices. Few functional traits exhibit low heritability and are susceptible to genotype–environment interactions; hence, cows selected for superior performance in conventional systems may not demonstrate equivalent genetic merit under organic management. Therefore, future studies should consequently proceed toward integration of genome, phenome and health across environments, representing Genomically Optimized Organic Dairy (GOOD). Through this GOOD model, breeding programs can combine genomic selection with precision phenotyping and health monitoring to simultaneously target disease resistance, fertility, longevity, milk quality and resilience to identify cattle that are better adapted and provide better performance to organic systems. Eventually, this GOOD framework provides a vision for the future of increasing organic dairy productivity while maintaining animal welfare, sustainability, and long-term food-system resilience.

## Data Availability

This is a review and all information here is from the published literature.

## Supplementary Materials

The following supporting information can be downloaded at: https://www.mdpi.com/article/10.3390/genes17090990/s1; Table S1: Regulation of organic farming in different countries; Table S2: Heritabilities; Figure S1: Numbers of organic dairy cows.
