# Supplementary material for: From Genome to Phenome: Genotype × Environment Interactions in Organic and Conventional Dairy Systems and the Emergence of Genomically Optimized Organic Dairy (GOOD)
Source: Genes (Basel). 2026 Aug 24;17(9):990. doi: 10.3390/genes17090990 (PMC13606531; doi:10.3390/genes17090990)

Figure S1: Number of organic dairy cows in the USA [21]

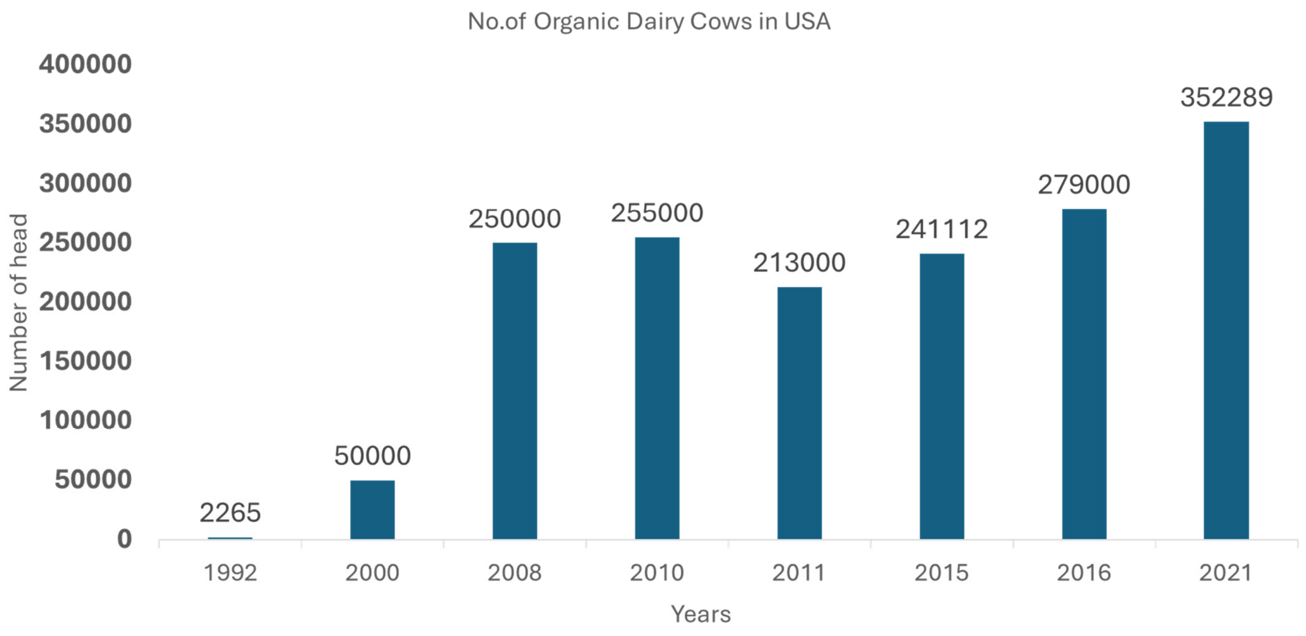

Supplement: Supplementary file 1 [file genes-17-00990-s001.zip › Figure S1 Numbers of Organic Dairy Cows.pdf]
