# Supplementary material for: From Genome to Phenome: Genotype × Environment Interactions in Organic and Conventional Dairy Systems and the Emergence of Genomically Optimized Organic Dairy (GOOD)
Source: Genes (Basel). 2026 Aug 24;17(9):990. doi: 10.3390/genes17090990 (PMC13606531; doi:10.3390/genes17090990)
Supplement: Supplementary file 1 [file genes-17-00990-s001.zip › Table S1 Organic Farming Policies and Regulations.pdf]

Table S1: Regulation of organic farming in different countries [12-21]

Category	Items	Requirements
Animal origin		United States, Canada, European Union, New Zealand: Animals should be raised continuously in organic conditions for at least 12 months.<br>Australia: Dairy cows from a conventional source should be raised 6 months to consider their milk as organic
Feeding	Land	United States, Canada: Prohibited substances (synthetic fertilizer, pesticides, etc) should not be applied to land for a period of three years before harvesting of the crop or for grazing.<br>European Union: Prohibited substances (synthetic fertilizers, pesticides, heavy metals, etc.) should not be used. Authorized compost is acceptable.<br>New Zealand: Authorized compost and fertilizer can be used.<br>Australia: Synthetic fertilizer should not be applied for at least one year. Only approved organic and natural sources can be used under certain conditions.
	Grazing in pasture	United States and Canada: Pasture throughout the entire grazing season, especially not less than 120 days per year. At least 30% of total forage intake should be from pasture.<br>European Union: Allowed to pasture for grazing during favorable conditions. About 60% of DM in daily rations consists of fresh or dried fodder, hay, or silage.<br>New Zealand: Grazing all the time. At least 50% of the feed comes from pasture

		Australia: Grazing in an organic rangeland area . 100% feed should be organic. If extreme condition (fire, flood) happened, feed permitted by can be provided with certain conditions to maintain organic status.
	Feed additives & supplements	United States: FDA-approved feed additives, vitamins, and minerals are approved. However, feed supplements and animal by-products are not allowed.<br><br>Canada: Vitamins approved by Canadian regulations are accepted. No Feed Additives and supplements are greater than those required at a certain age. No animal by-products.<br><br>European Union: Authorized feed additives are allowed. However, synthetic amino acids are not allowed.<br><br>New Zealand: Approved vitamins and minerals are allowed.<br><br>Australia: Minerals supplements can be given and feed supplements should be organic.
	GMO feeds	United States, Canada, European Union, New Zealand, Australia: GMO feeds are prohibited in every country.
	Calf management	United States: Milk replacers are prohibited.<br><br>Canada: Milk replacers from organic sources are used only in an emergency. Organic milk is allowed for up to 3 months.<br><br>European Union: Milk replacers are not allowed. Only organic milk can be provided.<br><br>New Zealand: Only Milk until 3 months of calves.<br><br>Australia: There is no available information about milk replacer.

Reproduction	Artificial insemination	United States, Canada, European Union, New Zealand, Australia: Approved in every country.
	Estrous synchronization	United States: Synthetic hormones cannot be used.<br><br>Canada, New Zealand, Australia: Synthetic hormones cannot be used.<br><br>European Union: Synthetic hormones cannot be used except for treatment purposes.
	Multiple ovulation an embryo transfer (MOET)	United States: Did not mention specifically about MOET.<br><br>Canada, European Union, New Zealand, Australia: Prohibited in every country.
	Animal cloning	United States, Canada, European Union, New Zealand, Australia: Prohibited in every country.
	Hormone	United States, Canada: Growth and breeding hormones cannot be used. Oxytocin is used only for postpartum therapeutics.<br><br>European Union: Growth hormone cannot be used.<br><br>New Zealand: Growth and breeding hormones cannot be used. Natural prostaglandin and oxytocin can be used in parturition cases.<br><br>Australia: Reproductive hormones cannot be used.
Welfare and health	Housing	United States: Continuous confinement of animals after 6 months of age is prohibited except for illness, parturition, and extreme weather. Bedding materials like straw, hay should be organic.

		Canada: comfortable surroundings, fresh air and opportunity for exercise. Bedding materials like straw, hay should be organic.<br><br>European Union: Litter-free housing prohibited.<br><br>New Zealand: Sufficient space and natural bedding materials should be provided.<br><br>Australia: Maintain adequate stocking density for allowing sufficient space for their natural movement. More than 16 hours of light should not be provided. Bedding materials like straw, hay should be organic sources.
	Pest management	United States, European Union, New Zealand, Australia: Not available information on pest management.<br><br>Canada: For fly control, sticky traps and traps baited with an attractant can be used.
	Disbudding or Dehorning	United States, New Zealand: There has not available information on disbudding or dehorning.<br><br>Canada, European Union, Australia: Acceptable upon permission from authority.
	Hoof trimming	United States, New Zealand: There has not available information.<br><br>Canada, European Union, Australia: Acceptable upon permission from authority.
	Tail docking	United States: Prohibited.<br><br>Canada, European Union, Australia: Acceptable upon permission from authority.

		New Zealand: There has not available information.
	Castration	United States, New Zealand: There has not available information.<br><br>Canada, European Union, Australia: Acceptable upon permission from the authority
	Antibiotic	United States, Canada, New Zealand, Australia: Prohibited. However, to reduce the suffering of animals, antibiotics may be used under a veterinarian's direction, but the product can't maintain its organic status.<br><br>European Union: Permitted under strict conditions (when phytotherapeutic, homeopathic options were not effective)
	Anthelmintics	United States: Prohibited slaughter stock, allowed in emergency treatment for dairy and breeder stock. Fenbendazole and Moxidectin can be used with the required withdrawal period.<br><br>Canada: synthetic parasiticides are prohibited except few approved drugs.<br><br>New Zealand: Synthetic parasiticides are prohibited.<br><br>European Union, Australia: There is no available information.
	Vaccine	United States, Canada, European Union, New Zealand, Australia: Approved vaccine can be applied.
	Prevention of disease	United States, Canada, European Union, New Zealand: There has not available information.<br><br>Australia: Breed selection, Grazing management, High-quality feed, Appropriate stocking density of animals.

	Treatment	United States: Electrolytes, topical treatment, external parasiticide or local anesthetic and aspirin are allowed.<br><br>Canada: Homeopathy, Ayurvedic and herbal products, acupuncture, trace elements or vitamins, probiotics, and local anesthetics are allowed.<br><br>European Union: Immunological veterinary medicines are allowed.<br><br>New Zealand: Natural purgatives and homoeopathic remedies, Zinc sulphate or copper sulphate are allowed.<br><br>Australia: Phytotherapeutic or homeopathic product can be used.
Regulation and references		United states: https://www.ecfr.gov/current/title-7/subtitle-B/chapter-I/subchapter-M/part-205/subpart-G/subject-group-ECFR0ebc5d139b750cd/section-205.603#p-205.603(d)(2).<br><br>Canada: Canadian Organic Standards - Organic Federation of Canada.<br><br>European Union: https://eur-lex.europa.eu/legal-content/EN/TXT/?uri= CELEX:32018R0848#d1e32-56-1.<br><br>New Zealand: https://www.biogro.co.nz/biogro-organic-standards.<br><br>Australia: https://www.agriculture.gov.au/sites/ default/files/documents/national-standard-edition.pdf. National Standard for Organic and Bio-Dynamic Produce, 2022.
